# Light, rather than circadian rhythm, regulates gas exchange in ferns and lycophytes

**DOI:** 10.1093/plphys/kiad036

**Published:** 2023-01-24

**Authors:** Daniela Aros-Mualin, Carmela Rosaria Guadagno, Daniele Silvestro, Michael Kessler

**Affiliations:** Department of Systematics and Evolutionary Botany, University of Zurich, Zurich 8008, Switzerland; Department of Botany, University of Wyoming, Laramie 82071, USA; Department of Biology, University of Fribourg, Fribourg 1700, Switzerland; Department of Biological and Environmental Sciences and Global Gothenburg Biodiversity Centre, University of Gothenburg, Gothenburg SE-405 30, Sweden; Swiss Institute of Bioinformatics, Fribourg 1700, Switzerland; Department of Systematics and Evolutionary Botany, University of Zurich, Zurich 8008, Switzerland

## Abstract

Circadian regulation plays a vital role in optimizing plant responses to the environment. However, while circadian regulation has been extensively studied in angiosperms, very little is known for lycophytes and ferns, leaving a gap in our understanding of the evolution of circadian rhythms across the plant kingdom. Here, we investigated circadian regulation in gas exchange through stomatal conductance and photosynthetic efficiency in a phylogenetically broad panel of 21 species of lycophytes and ferns over a 46 h period under constant light and a selected few under more natural conditions with day–night cycles. No rhythm was detected under constant light for either lycophytes or ferns, except for two semi-aquatic species of the family Marsileaceae (*Marsilea azorica* and *Regnellidium diphyllum*), which showed rhythms in stomatal conductance. Furthermore, these results indicated the presence of a light-driven stomatal control for ferns and lycophytes, with a possible passive fine-tuning through leaf water status adjustments. These findings support previous evidence for the fundamentally different regulation of gas exchange in lycophytes and ferns compared to angiosperms, and they suggest the presence of alternative stomatal regulations in Marsileaceae, an aquatic family already well known for numerous other distinctive physiological traits. Overall, our study provides evidence for heterogeneous circadian regulation across plant lineages, highlighting the importance of broad taxonomic scope in comparative plant physiology studies.

## Introduction

Photosynthetic organisms are characterized by an intrinsic circadian regulation, allowing biological processes to be temporally synchronized to daily and seasonal environmental cycles (Resco de Dios and Gessler, 2018). The early evolution of circadian regulation has been essential for plant adaptation on Earth and signs of it can be found in green algae (Nilsson, 2009). The resonance between the endogenous clock with the exogenous cycles has been widely shown to affect plant performance, especially for species living under more variable environmental conditions such as at high latitudes or under large diurnal temperature changes (Michael et al., 2003; Dodd et al., 2005; de Montaigu et al., 2015; de Montaigu and Coupland, 2017; Yari Kamrani et al., 2022). A broad range of physiological processes—ranging from RNA transcription to photosynthesis and hydraulics—shows circadian regulation (Hennessey and Field, 1991; Somers et al., 1998; Caldeira et al., 2014; Dodd et al., 2014). In particular, pronounced rhythms in stomatal conductance and photosynthesis have been found to be widespread in angiosperms (Resco de Dios and Gessler, 2018). Experimental evidence under free-running conditions, i.e. stable conditions with continuous light or darkness for at least 24 h, has revealed that 30%–53% of the fluctuation in stomatal conductance and 15%–25% in photosynthetic rate in angiosperms can be explained by internal rhythms (Resco de Dios and Gessler, 2018). This is advantageous for the plants because guard cells can take up to half an hour to react to sudden changes in environmental conditions, and an intrinsic stomatal regulation can optimize the efficiency of water use and carbon gain (Kübarsepp et al., 2020; Resco de Dios et al., 2020; Simon et al., 2020; Yari Kamrani et al., 2022). Available evidence indicates that photosynthesis is regulated by a clock that is independent from that of the stomata (Doughty et al., 2006; García-Plazaola et al., 2017; Resco de Dios et al., 2020), which is not surprising as photosynthesis is the result of several coordinated processes (light harvesting, electron transport, Rubisco activity, sugar production—in the Calvin cycle—and translocation), whereas stomata mainly affect net assimilation rate through the regulation of CO_2_ supply.

Although the clock represents a clear orchestrator for key plant processes (McClung, 2006), very little is known about the evolution of an internal circadian regulation for photosynthesis and stomatal conductance among the major lineages of land plants. A large body of literature covers the presence of circadian regulation in angiosperms, mainly focusing on model plants and small herbaceous species, such as Arabidopsis (*Arabidopsis thaliana*) and Turnip (*Brassica rapa)* (Dodd et al., 2004; Litthauer et al., 2015; Greenham et al., 2017; Resco de Dios and Gessler, 2018). However, to the best of our knowledge, there are only two studies of photosynthetic regulation in gymnosperms (Pavlovič et al., 2009; Gyllenstrand et al., 2014), none in lycophytes, and only one in ferns under constant light (Aros-Mualin et al., 2022). This lack of information on the evolution of circadian rhythms has become more concerning in the light of studies highlighting fundamental differences in the overall physiological responses between angiosperms and early divergent vascular plants (McAdam and Brodribb, 2012a, 2012b; Sussmilch et al., 2017; Kübarsepp et al., 2020; Gong et al., 2021; Cândido-Sobrinho et al., 2022).

Abscisic acid (ABA) and several clock-related genes, such as the pseudo-response regulator (PRR) gene family, are ubiquitous in the plant kingdom (Farré and Liu, 2013; Cai et al., 2017; Sussmilch et al., 2017). Lycophytes and ferns, however, show a passive response of stomatal opening to environmental changes, mainly driven by changes in their leaf water content during the day and without a clear effect of ABA on stomatal aperture (McAdam et al., 2016; Gong et al., 2021). Their responses to changes in CO_2_ concentration are slower than those of angiosperms and their photosynthetic capacity is highly reduced by distinct diffusional limitations (Carriquí et al., 2015; Kübarsepp et al., 2020). Thus, the presence of clock-related genes does not ensure a gate on the stomata behavior or photosynthesis of lycophytes and ferns. This leads to the current need to evaluate circadian regulation for stomatal conductance and photosynthesis throughout the land plant phylogeny to understand the evolution of circadian rhythms in photosynthetic organisms during the transition from water to land. More importantly, clarifying the possible diurnal gating for photosynthesis and stomatal behavior is central for implementing models to estimate broad-scale ecological processes. This is especially relevant considering the ecological relevance of ferns and lycophytes which can contribute to up to 70% of the local species richness in tropical floras (Kreft et al., 2010). Also, these plant groups dominated terrestrial ecosystems for much of the geological record (DiMichele and Phillips, 2002; Silvestro et al., 2015), so ecosystem-level processes in these plant communities might show fundamentally different patterns compared to today's angiosperm-dominated floras.

**Figure 1:**
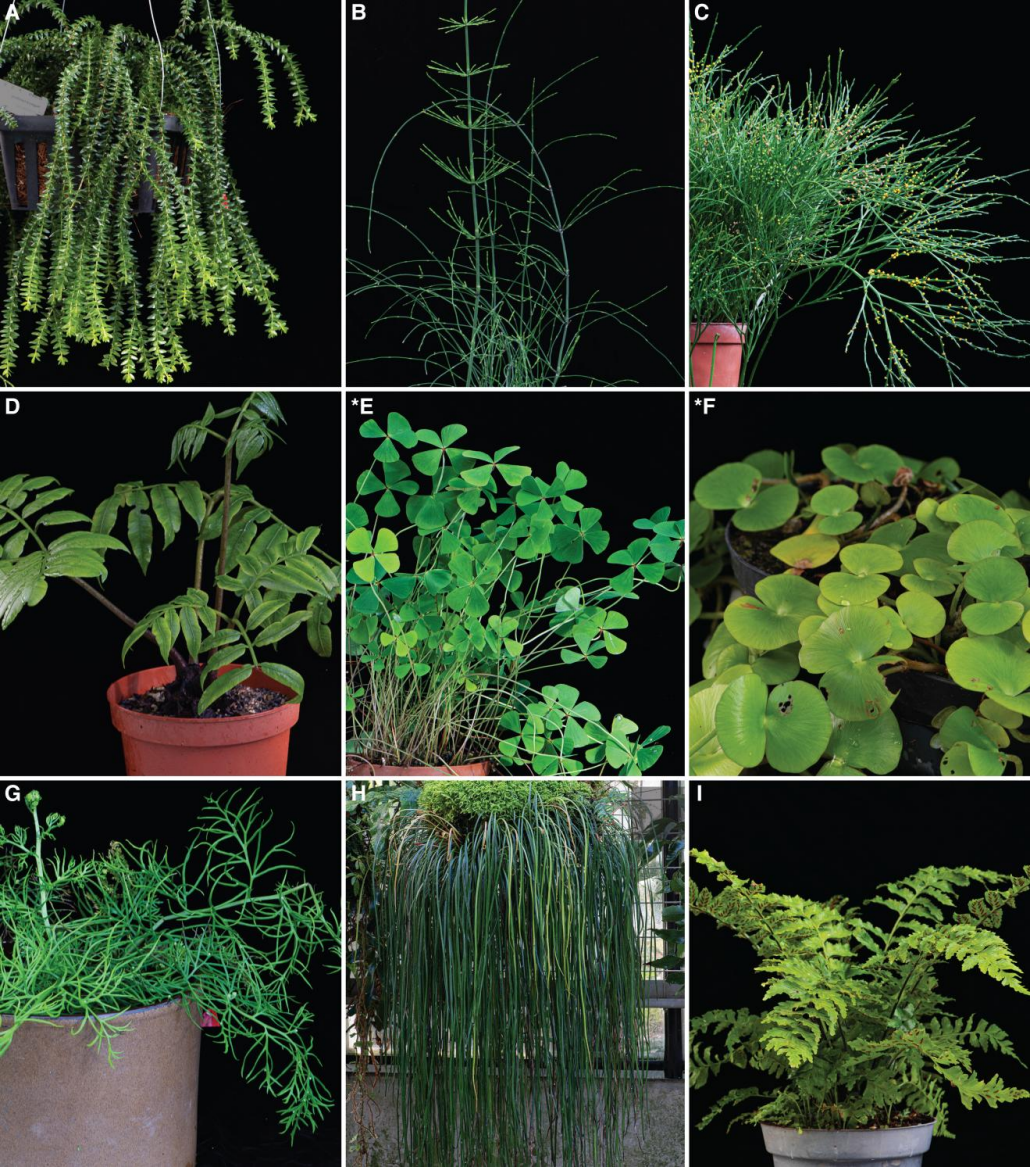
Morphological diversity among lycophytes and ferns included in the present study. The two species of Marsileaceae are marked with *. (A) Clubmoss (*Lycopodium hamiltonii*, Lycopodiaceae); (B) Horsetail (*Equisetum giganteum*, Equisetaceae); (C) Whisk fern (*Psilotum nudum*, Psilotaceae); (D) King fern (*Angiopteris evecta*, Marattiaceae); (E) Water-clover (*Marsilea azorica*, Marsileaceae); (F) 2-leaf water fern (*Regnellidium diphyllum*, Marsileaceae); (G) Water sprite (*Ceratopteris thalictroides*, Pteridaceae); (H) Shoestring fern (*Vittaria* sp., Pteridaceae); (I) Spleenwort fern (*Asplenium inaequilaterale*, Aspleniaceae). Photo credits: René Stalder.

Here, we set out to assess the occurrence of intrinsic circadian regulation in stomatal conductance and photosynthesis of lycophytes and ferns, uncovering its dependence on light–darkness queues to uphold a rhythm. To account for differences in habitats and evolutionary history, we included 19 species of ferns and 2 species of lycophytes, covering the major phylogenetic lineages in these groups (Table 1; Figure 1; Supplemental Figure 1). Moreover, we included species from a broad range of ecological conditions (tropical to temperate; aquatic to arid) that allow us to evaluate the dependence of circadian regulation on ecological background, as suggested for angiosperms (Doughty et al., 2006; Gil and Park, 2019). Through several time-course experiments, we monitored the diurnal responses under free-running conditions (46 h of continuous light) for all species to seek signs of periodicity of stomatal conductance, carbon assimilation, and photosystem II efficiency. Also, to assess the relative importance of internal clocks against external environmental cues, we further measured the diurnal stomatal behavior of selected fern species compared to angiosperms under variable greenhouse conditions.

**Table 1:** Species used in this study. Species included in the greenhouse experiment are marked with *. DT = desiccation-tolerant species

Species	Family	Order	Lifeform	Habitat
*Lycopodium hamiltonii* Spreng.	Lycopodiaceae	Lycopodiales	Epiphytic	Tropical
*Selaginella tamariscina* (P.Beauv.) Spring	Selaginellaceae	Lycopodiales	Terrestrial DT	Tropical
*Equisetum giganteum* L.	Equisetaceae	Equisetales	Terrestrial	Tropical
*Psilotum nudum* (L.) P.Beauv.	Psilotaceae	Psilotales	Epiphyte	Tropical
*Angiopteris evecta* (G.Forst) Hoffm.	Marattiaceae	Marattiales	Terrestrial	Tropical
*Osmunda claytoniana* L.	Osmundaceae	Osmundales	Terrestrial	Temperate
*Anemia phyllitidis* (L.) Sw.*	Anemiaceae	Schizaeales	Terrestrial DT	Tropical
*Marsilea azorica* Launert & Paiva	Marsileaceae	Salviniales	Semi-aquatic	Temperate
*Regnellidium diphyllum* Lindm.	Marsileaceae	Salviniales	Semi-aquatic	Tropical
*Sphaeropteris cooperi* (Hook. Ex F.Muell.) R.M.Tryon	Cyatheaceae	Cyatheales	Tree fern	Tropical
*Adiantum reniforme* L.	Pteridaceae	Polypodiales	Terrestrial	Temperate
*Ceratopteris thalictroides* (L.) Brongn.	Pteridaceae	Polypodiales	Aquatic	Tropical
*Oeosporangium chusanum* (Hook.) Fraser-Jenk.	Pteridaceae	Polypodiales	Terrestrial DT	Temperate
*Vittaria sp.*	Pteridaceae	Polypodiales	Epiphytic	Tropical
*Asplenium inaequilaterale* Willd.*	Aspleniaceae	Polypodiales (Eup. II)	Terrestrial	Tropical
*Diplazium proliferum* Brack.*	Athyriaceae	Polypodiales (Eup. II)	Terrestrial	Tropical
*Pseudophegopteris paludosa* (T.Moore) Ching.	Thelypteridaceae	Polypodiales (Eup. II)	Terrestrial	Tropical
*Dryopteris cambrensis* (Fraser-Jenk.) Beitel & W.R.Buck	Dryopteridaceae	Polypodiales (Eup. I)	Terrestrial	Temperate
*Tectaria zeilanica* (Houtt.) Sledge*	Tectariaceae	Polypodiales (Eup. I)	Terrestrial	Tropical
*Lecanopteris pumila* Blume	Polypodiaceae	Polypodiales (Eup. I)	Epiphytic	Tropical
*Polypodium vulgare* L.	Polypodiaceae	Polypodiales (Eup. I)	Epiphytic	Temperate
*Aframomum melegueta* (Roscoe) K.Schum.*	Zingiberaceae	Zingiberales	Terrestrial	Tropical
*Begonia mazae* Ziesenh.*	Begoniaceae	Cucurbitales	Terrestrial	Tropical
*Pellionia repens* (Lour.) Merr.*	Urticaceae	Rosales	Terrestrial	Tropical
*Strobilanthes persicifolia* (Lindl.) J.R.I.Wood*	Acanthaceae	Lamiales	Terrestrial	Tropical

## Results

### Growth chamber experiment

The diel light–dark cycles for five species showed the expected curve of increase during the morning and slow decrease after reaching its peak before the middle of the day, with changes more prominent for stomatal conductance than photosynthesis (Supplemental Figures 1–5). These data show that our approach is capable of measuring rhythmic changes in gas exchanges and that stomata can close in lycophytes and ferns during the night, although very slowly.

**Figure 2:**
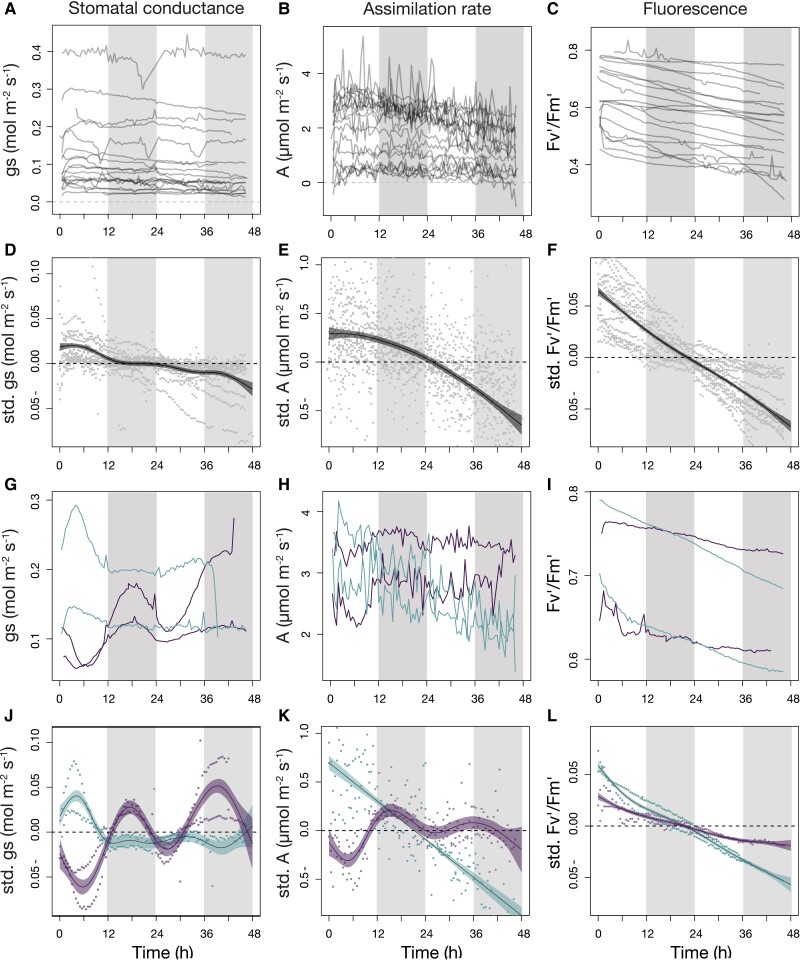
Gas exchange and photosynthesis over 46 h of free-running conditions from the growth chamber experiment. (A–C) and (G–I) shows the raw data measured against (D–F) and (J–L) zero-mean values without changing the original amplitude of the wavelength (std. as in standardized) of (A, D, G, J) stomatal conductance *gs*, (B, E, H, K) assimilation rate *A*, and (C, F, I, L) photosystem II operating efficiency (*F_v_′/F_m_′*). The lines in (D–F) and (J–L) represent generalized additive models (GAM) with the confidence intervals surrounding them in a lighter color. Gray and white areas represent subjective nights and days, respectively. (A–F), Overall lycophytes and ferns displayed in gray dots do not present a clear rhythm. (G–L) The species belonging to the Marsileaceae family are shown separately and are represented by water-clover (*Marsilea azorica*; dark purple) and two-leaf water fern (*Regnellidium diphyllum*; light green). Both species present a rhythm in *gs* (J), while water-clover also displays a minor rhythm in *A* (E).

In the main growth chamber experiment, stomatal conductance values ranged from 0.35 to 0.009 (mol H_2_O m^−2^s^−1^), assimilation rate from 5.36 to −0.89 (*µ*mol CO_2_ m^−2^ s^−1^), and photosystem II operating efficiency from 0.83 to 0.27 (Figure 2). The majority of fern and lycophyte species showed no indication of circadian rhythms in either stomatal conductance (*gs*) or photosynthesis measured as carbon assimilation rate (*A*) and the photosystem II operating efficiency (*F_v_′/F_m_′*) (Figure 2, D–F). Still, there were a few examples of a very weak rhythm in the stomatal conductance (Supplemental Figures 3 and 5) that may indicate a concealed circadian regulation. The generalized additive models, which were used to reveal nonlinear temporal trends, were a better fit in all cases than the linear models (Table 2). This was mainly driven by the expected increase of *gs* and *A* during the first 6 h of light, and their subsequent decay throughout the 2 days of constant light (Figure 2). These downward trends in both *gs* and *A* throughout the day are due to cumulative response to abiotic stress on the leaves induced by the free-running conditions (Guadagno et al., 2018). We also observed a subtle rhythm in the *F_v_′/F_m_′* of three species (Supplemental Figure 6), although the signal was so weak that it is not evident when all species are plotted together.

**Table 2:** Gas exchange of lycophytes and ferns under constant light described by GAMs and linear models. An individual model was applied for stomatal conductance (*gs*), assimilation rate (*A*), and photosystem II operating efficiency (*F_v_′/F_m_′*). Smaller values of the AIC and minimized GCV indicate the better-fitted models that are marked in italics and their differences found in ΔAIC and ΔGCV. If the differences are sizeble, then the values are marked in bold. *P* value of χ^2^ test comparing models. Significant values are marked *>0.001 **<0.001

Group	Variable	Model	AIC	ΔAIC	GCV	ΔGCV	*R* ^2^
All species except Marsileaceae	*gs*	GAM	*−8,006*.*1*		*−3,987*.*2*		*0*.232**
		Linear	−7,977.3	28.8	−3,967.6	19.6	0.214
	*A*	GAM	*1,699*.*8*		*855*.*4*		*0*.294**
		Linear	1,738.2	38.4	884.2	28.8	0.276
	*F_v_′’/F_m_′*	GAM	*−7,388*.*8*		*−3,683*.*5*		*0*.709**
		linear	−7,380.6	8.2	−3,669.4	14.1	0.707
Two-leaf water fern (*Regnellidium diphyllum*) Marsileaceae	*gs*	GAM	*−935*.*6*		*−449*.*7*		***0***.572**
		Linear	−852.4	**83**.**2**	−407.3	**42**.**4**	0.268
	*A*	GAM	*49*.*2*		*27*.*5*		*0*.693*
		Linear	49.2	0	38.2	10.7	*0*.*693*
	*F_v_′/F_m_′*	GAM	*−1,270*.*4*		*−620*.*5*		*0*.963**
		linear	−1,231.8	38.6	−594.7	25.8	0.952
Water-clover (*Marsilea azorica*) Marsileaceae	*gs*	GAM	*−879*.*6*		*−421*.*3*		***0***.747**
		linear	*−747*.*1*	**132**.**5**	*−355*.*4*	**65**.**9**	*0*.*446*
	*A*	GAM	*−105*.*5*		*−41*.*6*		***0***.407**
		linear	*−29*.*6*	**75**.**9**	*−0*.*7*	**40**.**9**	*0*.*054*
	*F_v_′/F_m_′*	GAM	*−1,338*.*9*		*−657*.*0*		*0*.852**
		linear	*−1,315*.*1*	23.8	*−636*.*2*	20.8	*0*.*826*

Conversely, two fern species of the family Marsileaceae (Figure 2, G–L) presented an evident rhythm in *gs*. The rhythm found for the two-leaf water fern (*Regnellidium diphyllum*) in *gs* did not translate into a rhythm for either *A* or *F_v_′/F_m_′*. However, the water-clover (*Marsilea azorica*) did present a mild rhythm in *A* that was absent in *F_v_′/F_m_′* and followed the same pattern as *gs*, suggesting ultimate stomatal aperture. The contrast of absence/presence of a rhythm is particularly notable when the ΔAIC (Akaike information criterion difference) and ΔGCV (generalized cross-validation difference) values between models are compared, together with the increase in explained variance (Table 2). It is also noteworthy that the rhythms of *the two-lead water fern* and *water-clover* were contrasting, with a peak during the day for the first species and during the night for the second one (Figure 2, G and J).

### Greenhouse experiment

**Figure 3:**
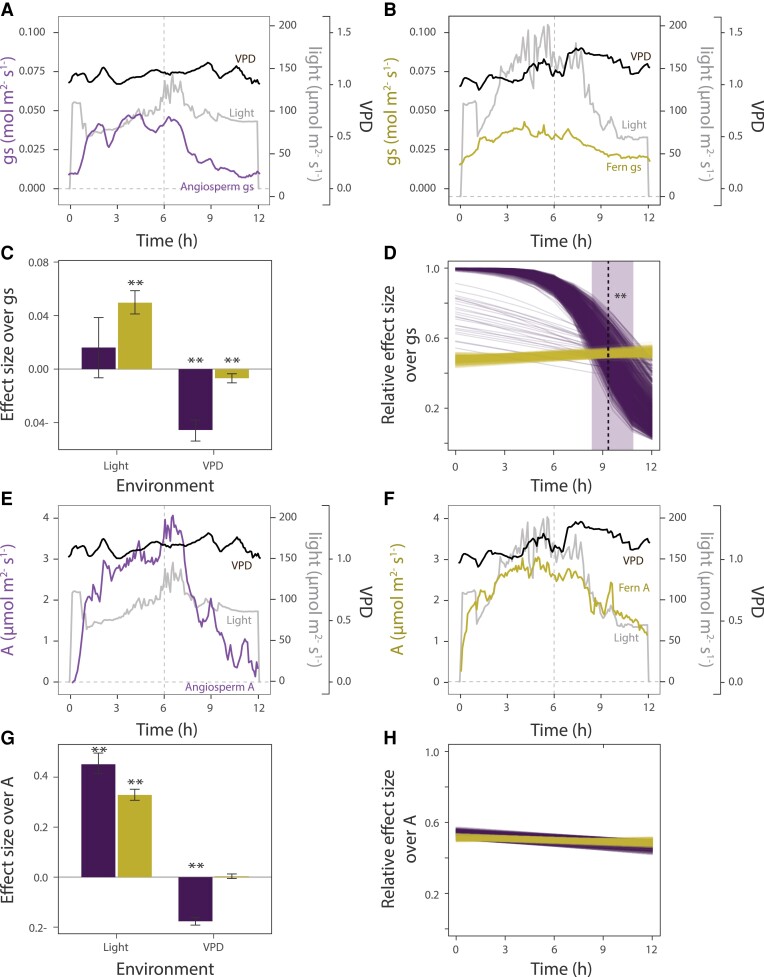
Stomatal conductance (*gs*) and assimilation rate (*A*) dynamics from the greenhouse experiment. Angiosperms are colored purple and ferns are yellow. Examples of raw *gs* and *A* measured during 12 h of light and later inputted to the Bayesian model are shown for one individual of (A and E) the angiosperm *Strobilanthes persicifolia* and (B and F) the Flowering fern (*Anemia phyllitidis*). The gray line represents the VPD and the black line the incident light in PPFD (light). In total, 4 replicates per species were used in the model. (C) and (G) show the correlation parameters obtained from de Bayesian model (Eqs 1 and 2) for light (ferns: 0.05 [CI: 0.04, 0.06], angiosperms: 0.02 [CI: −0.006, 0.04]), and VPD (ferns: −0.007 [CI: −0.01, −0.004], angiosperms: −0.046 [CI: −0.05, −0.04]). The bars represent the 95% credible interval (CI) of each variable and the correlation was considered significant when 0 was not included in the 95% CI. D and H, Stomatal opening and assimilation rate in dependence of the time of day (time) modeled as a logistic function. This result represents only 1 of 10,000 iterations from the total iterations used for the statistical analysis (1,000 lines per group in total). The plants had a 12 h light and 12 h darkness regime from 7 Am to 7 Pm. For ferns, time was not significant in regulating stomatal opening and assimilation rate. In angiosperms, for stomatal conductance, the mean steepness of the curve was −8.53 and the midpoint was at 9 h 25 min indicated by a vertical line with the confidence interval denoted by a light purple box. The dependence of the assimilation rate on time was not significant. Significant variables based on their 95% CI are marked with **.

In the greenhouse experiment, daily patterns were only observed in angiosperms and not in any of the measured ferns (Figure 3). In our Bayesian model, the presence of circadian regulation was described by a logistic function parameterized by the steepness *k*, and the midpoint *x_o_*, which indicates the time of day of inflection in the response. In angiosperms, *k* describing *gs* was −8.53 [95% credible interval, CI: −5.84, −11.62] and *x_o_* was 9 h 25 min [CI: 8 h 20 min, 10 h 53 min] for a 12/12 h day/night. This means that on average the stomata closed after 9 h 25 min of light (Figure 3D). The assimilation rate *A*, on the other hand, did not present a clear circadian pattern (Figure 3H), *k* was minimal with a value of 0.19 [CI: 0.07, 0.3], and the mean *x_o_* was at 12 h 32 min but with a credible interval essentially spanning the entire prior range (from 5 to 11 h 23 min after the start of day period) which makes the result not significant. Ferns did not show circadian regulation of either *gs* or *A* (Figure 3, D and H) because *k* was not significant (*gs*: 0.11 [CI: −0.04, 0.25]; A: 0.07 [CI: −0.02, 0.16]), meaning there was no steepness that could indicate a change.

Testing for the influence of environmental factors on *gs* (Figure 3, A–D; Supplemental Tables 1–4), we found a strong effect size of the incident light over the leaf (light) in ferns, showing that an increase in light led to increased stomatal aperture by an effect size of 0.05 [CI: 0.04, 0.06], whereas for angiosperms this relationship was not significant, since its estimated 95% CI overlapped with 0 (0.02 [CI: −0.006, 0.04]). Vapor pressure deficit (VPD) had the opposite effect, where higher VPD had a strong effect size over stomatal closure for angiosperms (−0.046 [CI: −0.05, −0.04]) but had a minimal impact for ferns (−0.007 [CI: −0.01, −0.004]). *A* showed a different pattern, presenting the highest correlation with light for both groups (ferns: 0.33 [CI: 0.31, 0.35]; angiosperms: 0.45 [CI: 0.42, 0.5]; Figure 3G). Ferns had no other significant factors affecting *A*. In angiosperms, *A* showed a moderate correlation with VPD (−0.18 [CI: −0.19, −0.16]).

## Discussion

In angiosperms, stomatal conductance and photosynthesis have been undoubtedly shown to have circadian regulation (Dodd et al., 2014; Hubbard and Webb, 2015). So far, no studies have assessed circadian rhythms in lycophytes and only one study exists for ferns under sustained constant light (Aros-Mualin et al., 2022). Here, for the majority of fern and lycophyte species in the experimental panel, we found no patterns of circadian dynamics in gas exchanges after 12 h of constant light, except for a few very weak signals in some of the species and a distinct rhythm in stomal conductance for 2 members of the family Marsileaceae (Figure 2).

Our experimental panel, although limited to 21 of the 12,000 existing species, was carefully selected to cover the most important phylogenetic lineages across the ferns and lycophyte world, a wide range of habitats, and lifeforms. However, in order to avoid generalized assumptions, we must acknowledge the possibility that our failure to detect extended and distinct rhythms across the panel might be a result of the selected experimental conditions. It has already been shown that free-running conditions can potentially suppress circadian regulation in *Drosophila* (Yoshii et al., 2005), while drought conditions might induce stronger circadian regulation in *Arabidopsis thaliana* (Caldeira et al., 2014). Nevertheless, we remain confident in our experimental findings, especially for the robustness of the diel measurements for 5 of the 21 study species in the first experiment showing very distinct changes between day and night, confirming that our experimental setup was suitable to detect such rhythms, if present (Supplemental Figure 1). Moreover, for the two species of Marsileaceae, we detected clear rhythms during the free-running conditions (Figure 2), so there is no effective reason to think that the chosen conditions would not allow for detections in other species if present. Finally, under variable environmental conditions, we found clear circadian regulation in the four angiosperm species, but not in the simultaneously studied ferns, which was supportive, once again, of the ability of our experimental setup to detect rhythms (Figure 3). This latter experiment is particularly informative since it was conducted under common greenhouse conditions at which all species are thriving and healthy. Therefore, if circadian rhythms were to be found under a different set of climatic conditions, these would likely be less optimal and more stressful, rather than typical for the species.

Our study suggests then, that rhythms on gas exchanges in fern and lycophyte species might be largely triggered and maintained by regular external light cues—diel cycle conditions—and, unlike the case of many angiosperms, they are not regulated by internal circadian clocks.

### Circadian regulation of stomatal movement

In angiosperms, circadian regulation of stomatal movement is thought to result from the reciprocal interaction between the protein TOC1 (TIMING OF CAB EXPRESSION 1) and the hormone ABA (Legnaioli et al., 2009). TOC1 affects ABA concentrations by inhibiting the expression of ABA receptor proteins and allowing the stomata to open. In turn, ABA and other clock-related genes and proteins, such as the MYB transcription factors, will induce TOC1. This feedback loop ensures that the maximum sensitivity to ABA will be in the subjective afternoon, leading to stomatal closure (Hubbard and Webb, 2015). Although the essential machinery for the described circadian regulation is found in *Selaginella moellendorffii* (Farré and Liu, 2013; Bueno et al., 2019), there is no consensus on the sensitivity of ferns and lycophytes to ABA (e.g. Cai et al., 2017; Sussmilch et al., 2019). The ongoing debate is driven by the presence of several components of the ABA pathway (Cai et al., 2017) while simultaneously obtaining contrasting results to experimental ABA application (Brodribb and McAdam, 2011; Ruszala et al., 2011; McAdam and Brodribb, 2012a, 2012b; Hõrak et al., 2017; Cardoso et al., 2019; Cardoso and McAdam, 2019; Westbrook and McAdam, 2020a). Gong et al. (2021) found that ferns and lycophytes lack the synthesis of nitric oxide in the presence of ABA, a crucial piece in the ABA-signaling pathway responsible for stomatal closure. Additionally, Lima et al. (2019) found that ferns allocate more carbon to secondary compounds than angiosperms during diel measurements, proposing that higher accumulation leads to lower stomatal conductance and concluding that ferns and angiosperms may have fundamentally different metabolisms. They also found similar rhythms in stomatal conductance and carbon assimilation to our results during the first 12 h of constant light, increasing over the morning and decreasing after midday. The accumulation of sugars and secondary compounds could be behind the continuous decrease of stomatal conductance and photosynthesis after the first subjective day of our study, although it may also result from other cumulative abiotic stress on the leaves induced by the free-running conditions. More importantly, we speculate that any potential rhythm is highly dependent on external triggers.

Marsileaceae species present an interesting case study since they show circadian regulation on stomatal conductance (Figure 2; Aros-Mualin et al., 2022) but seem to be insensitive to ABA (Westbrook and McAdam, 2020a). A key difference with circadian regulation present in angiosperms is the lack of stomatal closure under light conditions, where stomatal conductance only gradually increased or decreased over several hours. This could be explained by: (1) alternative independent-ABA-signaling pathways driving the rhythm or (2) slow changes in their conductance induced directly or indirectly by ABA. Westbrook and McAdam (2020a) found no differences in the stomatal conductance of two Marsileaceae species after 30 min of administering ABA. Since the changes we found are over the course of several hours, it could be that more time is needed to find an effect. Still, in a separate study, we found circadian regulation in the leaf movement of two water-clover species (*Marsilea*) that could be linked with stomatal conductance (Aros-Mualin et al., 2022). Leaf movement is driven in a similar manner to that of the bean pulvinus, which is regulated by water movement and ions (Irving et al., 1997; Kao and Lin, 2010). Therefore, osmotic regulation arises as a compelling explanation for both rhythms, although alternative explanations cannot be discarded without more in-depth studies.

**Figure 4:**
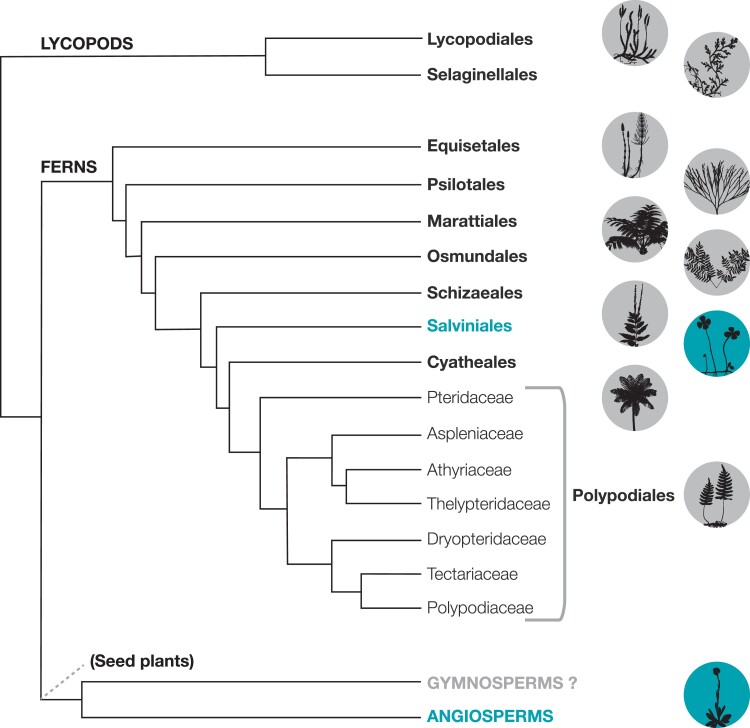
Simplified phylogeny of vascular plants emphasizing the presence of circadian rhythms in gas exchange among plant groups studied here. The two groups with marked circadian rhythms in stomatal conductance are highlighted in light blue, whereas in black (or gray background) are all the orders and major clades that did not present a clear rhythm. Gymnosperms are marked in gray and with a question mark because there is not enough information in this respect. Adapted from the PGG I (2016) fern phylogeny.

Nevertheless, the absence of clear rhythm after the first 12 h found by us among most lycophytes and ferns suggests that circadian regulation over stomatal control could either have been acquired de novo among Marsileaceae, along with the numerous other unusual stomatal responses (Figure 4), or that it depends on alternative pathways to sustain the rhythm for longer without external cues. Both are plausible explanations since their unique physiology allows Marsileaceae to have some of the highest assimilation rates known among ferns (Wu and Kao, 2011), to open stomata in the presence of blue light (Westbrook and McAdam, 2020b), to be the only ferns known to have wrong-way stomatal responses (Westbrook and McAdam, 2020a) and phototropism (Gómez, 1981; Kao and Lin, 2010), with water-clover species also having nyctinastic movements (Minorsky, 2019). In all of this, Marsileaceae resembles angiosperms more closely than other ferns, which may have paved the way for the development of circadian regulation in stomatal movement. It is conceivable that this is due to the semi-aquatic lifestyle of this family, which created a set of physiologically stressful conditions that are atypical for ferns (Aros-Mualin et al., 2022). Interestingly, in our study, water sprite fern (*Ceratopteris thalictroides*), which also grows under variable aquatic conditions, did not show a rhythm in gas exchange under free-running conditions.

### Circadian regulation of photosynthesis

Due to the complexity of the overall photosynthetic pathway, with a series of processes carried out in different parts of the leaf, from the stomata to the chloroplasts, the study of circadian regulation for carbon assimilation and utilization has been far more challenging than that for stomatal conductance, and, therefore, it is poorly understood even for angiosperms (Resco de Dios and Gessler, 2018). This means that interpreting the lack of circadian regulation in photosynthesis can prove challenging (Figure 2). Cuitun-Coronado et al. (2022) found clear indications of circadian regulation over the photosynthetic state of photosystem II in *Marchantia polymorpha*, a liverwort that shares some components of the circadian oscillator with flowering plants (Linde et al., 2017). Our findings for ferns and lycophytes show no rhythm in the photosystem II operating efficiency or in the carbon assimilation rate that closely follows the stomatal conductance pattern. This result leads us to believe that circadian regulation was either lost for members of these two groups or suppressed under free-running experimental conditions.

Nonetheless, we propose that having a strong circadian regulation in photosynthesis may not provide a clear advantage for ferns or lycophytes. Their assimilation rate values are generally lower than those found in seed plants, primarily due to diffusional limitations on mesophyll conductance (Tosens et al., 2016). They also have smaller surfaces of chloroplasts facing the intercellular air space, and, at the same time, thicker cell walls when compared to angiosperms (Tosens et al., 2016). Since leaf respiration rates in ferns and lycophytes are similar to angiosperms, they have a much lower carbon balance at the leaf level, which translates into an overall lower photosynthetic capacity (Carriquí et al., 2015). Additionally, photosystem II operating efficiency (*F_v_′/F_m_′*) depends on the gene expression of the light-harvesting complex proteins (*lhc* mRNA). Diurnal patterns in *lhc* mRNA in angiosperms are thought to result from the light-dependent pathway for chlorophyll biosynthesis (Oberschmidt et al., 1995). The presence of a light-independent pathway for chlorophyll in ferns and lycophytes, present also in gymnosperms but not angiosperms (Armstrong, 1998), could be behind the absence of a rhythm in *F_v_′/F_m_′* under these experimental conditions.

### Implications for fern and lycophyte ecology

Our study raises the question of whether the absence of circadian regulation would be functionally meaningful in providing any ecological advantage to ferns and lycophytes. Although the patterns found by us under artificial conditions do not rule out that circadian rhythms may be more pronounced among ferns and lycophytes under natural or more stressful conditions, they suggest that stomatal regulation is mainly determined by external cues. This may have two reasons. On the one hand, as detailed above, ferns and lycophytes have several physiological limitations compared to angiosperms that may reduce the advantages of circadian regulation. On the other hand, they may lack key regulatory processes such as ABA-mediated responses. In this scenario, it is possible that for ferns to remain competitive, they need to be photosynthetically active whenever there is light available (Creese et al., 2014). The results from our greenhouse experiment with variable environmental conditions are in line with this hypothesis (Figure 3), with ferns showing that their stomatal conductance and photosynthetic rates depended exclusively on light availability, whereas in angiosperms they were largely regulated by VPD along with their pronounced circadian regulation. Responses of fern stomata to VPD are thought to be directly related to the water status of the leaf cells (McAdam and Brodribb, 2015). In other words, as long as ferns have enough water available in the soil and leaf tissue, stomata will remain open despite changes in incoming radiation or VPD (Creese et al., 2014). Under such conditions, there may simply be no advantage to developing complex circadian regulation mechanisms.

The majority of fern and lycophytes species grow in humid and shady environments (Kessler et al., 2011; Weigand et al., 2020), but some species also grow in sunny, exposed habitats and even in arid regions. We propose that if marked circadian regulation occurs in ferns other than Marsileaceae, it would most likely be found in species from habitats more exposed to light. However, our growth chamber study included three desiccation-tolerant species and five epiphytic species (Table 1; Figure 1, C and H) that are more susceptible to drought than soil-rooted species. Still, no clear indication of circadian regulation was found for any of the measured physiological traits. Although more extensive sampling is needed for a complete understanding of rhythms in ferns and lycophytes, the present results suggest that drought-prone environmental conditions for growth do not dictate the presence or absence of circadian regulation in these two groups.

### Conclusions

The lack of a clear intrinsic rhythm in gas exchanges of our experimental panel of ferns and lycophytes is suggestive of a circadian regulation being highly light dependent in these groups. Most importantly, circadian regulation does not cause sudden or complete stomatal closure in any ferns studied, unlike angiosperms. The Marsileaceae family, an evolutionarily old semi-aquatic group, stood out showing an evident rhythm in stomatal conductance. The mechanisms driving stomatal movement in Marsileaceae present a unique experimental opportunity to unravel the selective pressure for the development of circadian regulation and new pathways for stomatal control in an evolutionarily independent case from angiosperms. Our study adds to the discussion of the dissimilarities in stomatal responses among lycophytes, ferns, and angiosperms, and it places previous knowledge of circadian regulation in a broader evolutionary context. In the future, possible energetic disadvantages in developing an internal rhythm should be taken into account for species other than angiosperms, and the inclusion of lycophytes and ferns in comparative studies of plant physiology will provide substantial added value.

## Materials and methods

### Plant material

We selected 2 species of lycophytes and 19 species of ferns to have a broad representation of the diversity of seedless vascular plants (Table 1, Figure 1). We included members for most orders of these two major lineages (3 and 11 orders, respectively; Supplemental Figure 7) except for Isoëtales within the lycophytes because they are underwater plants, Ophioglossales and Gleicheniales among ferns because of their challenging cultivation, and Hymenophyllales because of their lack of stomata. Since Polypodiales is a large order comprising more than 80% of fern biodiversity (>9,500 species; PPG I, 2016), we incorporated representatives from three major clades within this order: Pteridaceae, eupolypods I, and eupolypods II. Species were also selected to cover a wide range of ecological types, including different lifeforms (terrestrial, epiphytic, and semi-aquatic) and different habitats from tropical to temperate. The 4 angiosperm species used for comparison were all tropical and distributed throughout the phylogeny, including a monocot (Zingiberales) and three eudicots (fabid and lamiid clades). All plants used for the experiment reported here were fully mature and cultivated in the Botanical Garden of the University of Zurich, Switzerland.

### Experimental setup

We conducted two sets of experiments. The first experiment was under free-running conditions in growth chambers (Versatile Environmental Test Chamber MLR-351, Sanyo Electric Co., Osaka, Japan). Plants were first acclimated for 2 weeks using a 12 h photoperiod with 80% relative humidity (RH), temperature set at 19°C, and 50 *µ*mol m^−2^ s^−1^ of light. These conditions were chosen based on recommendations of the gardeners from the Zurich Botanical Garden regarding the average optimal conditions for the cultivation of the selected species without causing substantial stress. After the acclimation period, plants were transferred to a measuring growth chamber with identical conditions but constant light for 46 h (free-running conditions).

The second experiment aimed at testing the findings of the first experiment under more variable conditions and comparing ferns and angiosperms. It was performed inside a greenhouse where the environment was controlled to imitate tropical conditions but allowed natural light due to a glass ceiling. Because light and temperature conditions varied greatly between sunny and cloudy days, there was no control over the maximum temperature value, light, or RH. The values ranged between 18°C (min) and 35°C (max), 70 *µ*mol m^−2^ s^−1^ and 230 *µ*mol m^−2^ s^−1^ photosynthetic photon flux density (PPFD), and 80% and 100% RH. The experiment was conducted during February 2021 (winter, light period of 8 h), so lamps provided additional lighting of 70 *µ*mol m^−2^ s^−1^ from 7 Am to 7 Pm to keep a 12 h photoperiod. We used four tropical fern and angiosperm species each distributed throughout the phylogeny (Table 1) with three replicates *per* species of the same age and size. All plants were previously acclimated for a month to the greenhouse conditions before starting the measurements and were fully watered every 2 days for its duration.

### Measurements

Continuous gas exchange measurements were conducted over 46 h for the growth chamber experiment and 12 h for the greenhouse experiment. In both cases, measurements were obtained using a portable infrared gas analyzer to measure leaf gas exchange (LI6400XT, LICOR Inc., Lincoln, NE, USA) with the Auto-Log function. In the growth chamber experiment, we used a fluorometer head (LI6400-40, LICOR Inc., Lincoln, NE, USA) to include fluorescence measurements, whereas, in the greenhouse experiment, we used a transparent head to allow natural light to reach the leaf.

For the growth chamber experiment, assimilation rate (*A*), stomatal conductance (*gs*), and photosystem II operating efficiency (*F_v_′/F_m_′*) were recorded every 30 min using the Autolog function following established methods (Long and Bernacchi, 2003) and keeping the conditions inside the chamber of the portable photosynthesis system as close as possible to the external environment (19°C, 70 PPFD, 75% RH). To ensure that our experimental approaches are sensitive enough to detect rhythmic activity, we also measured diel rhythms in five species listed below for 48 h, adapted to 12 h light–dark cycles: Flowering fern (*Anemia phyllitidis*), Spleenwort fern (*Asplenium inaequilaterale*), Water sprite (*Ceratopteris thalictroides*), Adders fern (*Polypodium vulgare*), and Spike-moss (*Selaginella tamariscina*).

For the greenhouse experiment, *A*, *gs*, the incident light, and VPD were recorded every 5 min. Because the temperature outside the portable photosynthesis system was variable throughout the day while being kept constant at 25°C inside the chamber, the RH and, therefore, VPD in the chamber varied greatly. Additionally, we changed the RH manually twice a day (2 h after sunrise and at around 2 Pm) to increment variation in VPD conditions. No fluorometer was used during the greenhouse experiment to allow for chambers conditions to match the sudden variations in incoming radiation.

### Statistical analysis

#### Growth chamber experiment

Because the different study species varied enormously in their magnitude of *A*, *gs*, *F_v_′*/*F_m_′* (Figure 2, A–C), and since we did not conduct analyses on a per-species basis (except for Marsileaceae), we normalized data across species to search for patterns. For this, we subtracted the mean values obtained throughout the 46 h of constant light *per* species to have all species with zero means and examined temporal variations of the three variables (*A*, *gs*, and *F_v_′*/*F_m_′*) with generalized additive models (GAM) fitted with automated smoothness selection. We compared the GAM precision to a linear model with an ANOVA and used the AIC together with the minimized generalized GCV values. All calculations were done with the “mgcv” library in the R software environment (Wood, 2011; R Core Team, 2021).

#### Hierarchical Bayesian regression for greenhouse experiment

The aim of this analysis was to disentangle the relative influences of relative air humidity and light availability (which varied in the greenhouse) against the time of day as an indicator of circadian regulation in determining *gs* and *A*. We used multiple regression analysis assuming that *gs* and *A* respond linearly to the environmental variables they are exposed to (*light* and *VPD*) and logistically to the time of the day (*t*). The logistic function was used to assess the presence of circadian rhythms and if there is a point in time where *gs* is reduced or *A* stops regardless of changes in the environmental variables. Circadian rhythms are better described by sinusoid shapes when the whole period is considered. However, since we only measured half a period (only 12 h corresponding to daytime), periodicity is lost, so a logistic function becomes a better fit.

Thus, for an individual of species *sp*, the expected *gs* and *A* at time *t* are:


(1) $${\bar{gs}}_{t}=\gamma_{sp}+a_{1}\times light_{t}+a_{2}\times VPD_{t}+a_{3}\times \frac{1}{1+e^{k(t-x_{0})}}$$

(2) $${\bar{A}}_{t}=\alpha_{sp}+b_{1}\times light_{t}+b_{2}\times VPD_{t}+b_{3}\times \frac{1}{1+e^{k(t-x_{0})}}$$

where the vectors of coefficients *a* = {a_1_, a_2_, a_3_} and *b* = {b_1_, b_2_, b_3_} and the parameters of the logistic function (the midpoint *x*_0_ and the steepness *k*) are shared across all individuals and species of the same pylogenetic major group (estimated independently for angiosperms and ferns). The model also includes species-specific intercepts (γ_sp_ and α_sp_) shared across individuals of the same species. Based on this model, we modeled the measured *gs* and *A* to be normally distributed around their expected value:


(3) $$gs∼N(\bar{gs},\;\sigma_{gs})\;\mathrm{and}\;A∼N(\bar{A},\;\sigma_{A})$$

where *σ*_*gs*_ and *σ*_*As*_ are species-specific random errors, shared across all individuals of each species.

We implemented our model in a Bayesian framework to jointly estimate the linear coefficients, parameters of the logistic, intercepts, and random errors across all individuals and species in each group. We, therefore, used a normal density to compute the likelihood of the measured *gs* and *A* based on Equation (3) and defined prior distributions for all parameters. Specifically, we used normal prior distributions for the coefficients *a* and *b* centered in 0 and with a standard deviation of *σ* ($a∼N(0,\;\sigma_{a})$ and $b∼N(0,\;\sigma_{b})$), a normal distribution for k∼N(0,3), and a uniform distribution for $x_{o}∼U(0,1)$. We considered the standard deviations of the priors on *a* and *b* as unknown and assigned them an exponential hyper-prior $\sigma_{a},\;\sigma_{b}\;∼\mathrm{Exp}(1)$. Similarly, we used gamma distributed priors on the random effects for each species $\sigma_{gs}∼\Gamma (\alpha_{1},\;\beta_{1})$ and $\sigma_{As}∼\Gamma (\alpha_{2},\;\beta_{2})$, with the shape and rate parameters themselves considered as unknown and assigned an exponential hyper-prior $\alpha_{1},\;\alpha_{2},\;\beta_{1},\;\beta_{2}∼\mathrm{Exp}(0.1)$. The use of exponential hyper-priors on the standard deviation of the normal priors implies that all else being equal, the model favors effect sizes narrowly distributed around zero. Thus, the use of hyper-priors allows us to control for over-parameterization by favoring shrinkage around the null hypothesis of 0-effects, while reducing the subjectivity of priors set on the parameters of interest (Gelman et al., 2013).

We estimated all model parameters together using Metropolis–Hastings Markov Chain Monte Carlo (MCMC). We used a sliding window proposal for all effect sizes and multiplier proposals for all parameters with a gamma distribution. We ran the MCMC for 1,000 M iterations, sampling every 100. We assessed the convergence of the chain by inspecting the trace and the effective sample sizes of the posterior and the parameter of interest in Tracer (Rambaut et al., 2014). We summarized the sampled parameter values by calculating their mean and 95% CIs, and we considered the correlation parameters as significant when 0 was not included in the 95% CI.

## Supplementary Material

kiad036_Supplementary_Data

## Supplemental data

The following materials are available in the online version of this article.

**Supplemental Table S1.** Raw data obtained from Tracer (Rambaut et al., 2014) for ferns’ stomatal conductance (*gs*).

**Supplemental Table S2.** Raw data obtained from Tracer (Rambaut et al., 2014) for angiosperms’ stomatal conductance (*gs*).

**Supplemental Table S3.** Raw data obtained from Tracer (Rambaut et al., 2014) for ferns’ assimilation rate (*A*).

**Supplemental Table S4.** Raw data obtained from Tracer (Rambaut et al., 2014) for angiosperms’ assimilation rate (*A*).

**Supplemental Figure S1.** Gas exchange measurements for a Flowering fern (*Anemia phyllitidis*) under diel light/dark cycles versus constant light.

**Supplemental Figure S2.** Gas exchange measurements for a Spleenwort fern (*Asplenium inaequilaterale*) under diel light/dark cycles versus constant light.

**Supplemental Figure S3.** Gas exchange measurements for a Water sprite (*Ceratopteris thalictroides*) under diel light/dark cycles versus constant light.

**Supplemental Figure S4.** Gas exchange measurements for an Adders fern (*Polypodium vulgare*) under diel light/dark cycles versus constant light.

**Supplemental Figure S5.** Gas exchange measurements for a Spike-moss (*Selaginella tamariscina*) under diel light/dark cycles versus constant light.

**Supplemental Figure S6.** Photosynthetic reaction centre rhythm of three fern species over 46 h of constant light.

**Supplemental Figure S7.** Phylogeny of vascular plants used in this study.
