# Supplementary material for: Light, rather than circadian rhythm, regulates gas exchange in ferns and lycophytes
Source: Plant Physiol. 2023 Jan 24;191(3):1634–47. doi: 10.1093/plphys/kiad036 (PMC10022864; doi:10.1093/plphys/kiad036)
Supplement: kiad036_Supplementary_Data [file kiad036_supplementary_data.pdf]

Supplemental data

Supplemental Table S1. Raw data obtained from Tracer (Rambaut *et al*., 2014) for ferns' stomatal conductance (gs). Four species were input in the model for estimating the overall effect of light, vapor pressure deficit (VPD) and circadian regulation (time), with independent intercepts (μ) and random error (σ) for each species. The hyper-priors for the random errors were α and β. Time is described by k and xo that are the parameters of a logistic function. The model had 1.000M iterations, but saving 1 each 100, and a burn-in of 10M.

**Variable**	**Mean**	**SE**	**SD**	**95% HPD int**	**ACT**	**ESS**
Posterior	4399.1637	0.0537	2.9537	4393.49, 4404.46	2.973E5	3027.2
Likelihood	4405.0081	0.0437	2.4153	4400.14, 4409.07	2.9512E5	3049.6
Prior	-5.8444	0.0248	1.6665	-9.1141, -3.5543	1.9914E5	4519.5
μ1	0.1059	6.92E-5	3.3E-3	0.1007, 0.1125	3.9654E5	2269.6
μ2	0.484	1.06E-4	5.8E-3	0.4723, 0.4949	2.9971E5	3002.9
μ3	0.0719	7.25E-5	3.5E-3	0.0622, 0.0784	3.9512E5	2277.8
μ4	0.1274	7.48E-5	3.6E-3	0.1212, 0.1339	3.8659E5	2328
σ1	0.00699	5E-6	2.6E-4	6.5E-3, 7.5E-3	3.3869E5	2657.3
σ2	0.1013	6.4E-5	3.4E-3	0.0956, 0.1087	3.2305E5	2786
σ3	0.0185	8.1E-6	5.6E-4	0.0174, 0.0196	1.8465E5	4874
σ4	0.0343	1.9E-5	1.1E-3	0.0322, 0.0364	2.9489E5	3052
**Light**	**0.0496**	**8.5E-5**	**4.4E-3**	**0.0412, 0.0586**	**3.3275E5**	**2704.7**
**VPD**	**-0.0068**	**3.5E-5**	**1.8E-3**	**-0.0103, -0.0035**	**3.572E5**	**2519.6**
**time**	**-0.0633**	**1.3E-4**	**6.3E-3**	**-0.0748, -0.0531**	**3.9852E5**	**2258.3**
σ-gs	0.0888	7.6E-4	0.0824	0.0211, 0.2094	76692.3199	11735.2
k	***-0.1102***	***1.2E-3***	***0.0723***	***-0.2484, 0.0364***	***2.4381E5***	***3691.4***

**xo**	**0.4997**	**5.6E-3**	**0.2654**	**0.0688, 0.9758**	**3.938E5**	**2285.4**
α	0.9931	8.1E-3	0.4871	0.2009, 1.9573	2.472E5	3640.7
β	19.147	0.2136	11.2125	1.638, 42.0088	3.2656E5	2756

9

Supplemental Table S2. Raw data obtained from Tracer (Rambaut *et al*., 2014) for angiosperms' stomatal conductance (gs). Four species were input in the model for estimating the overall effect of light, vapor pressure deficit (VPD) and circadian regulation (time), with independent intercepts (μ) and random error (σ) for each species. The hyper-priors for the random errors were α and β. Time is described by *k* and *xo* that are the parameters of a logistic function. The model had 1.000M iterations, but saving 1 each 100, and a burn-in of 10M.

**Variable**	**Mean**	**SE**	**SD**	**95% HPD int**	**ACT**	**ESS**
Posterior	3960.1487	0.0471	3.0367	3454.08, 3965.42	2.163E5	4160.8
Likelihood	3968.9139	0.0499	2.8979	3963.15, 3974.06	2.6691E5	3371.9
Prior	-8.7652	0.0348	2.1827	-12.95, -5.11	2.291E5	3928.3
μ1	0.0643	5.053E-5	2.6E-3	0.0593, 0.0694	3.3814E5	2661.6
μ2	0.0727	5.67E-5	3.02E-3	0.0669, 0.0787	3.1775E5	2832.4
μ3	0.0614	5.29E-5	2.6E-3	0.0563, 0.0663	3.7443E5	2403.6
μ4	0.0915	6.74E-5	3.5E-3	0.0845, 0.0979	3.3921E5	2653.2
σ1	0.0195	1.14E-5	7.5E-4	0.0181, 0.021	2.1042E5	4277.2
σ2	0.031	1.96E-5	1.1E-3	0.0287, 0.0332	2.646E5	3401.3
σ3	0.0143	7.75E-6	5.8E-4	0.0132, 0.0154	1.6365E5	5499.6
σ4	0.0566	1.81E-5	1.6E-3	0.0535, 0.0597	1.1445E5	7863.8
Light	***0.0161***	***2.28E-4***	***0.0115***	***-0.0065, 0.0385***	***3.5579E5***	***2529.6***
**VPD**	**-0.0455**	**8.02E-5**	**4E-3**	**-0.0538, -0.0381**	**3.6136E5**	**2490.6**
**time**	**-0.0344**	**1.22E-4**	**6.4E-3**	**-0.0479, -0.0238**	**3.2231E5**	**2792.4**
σ-gs	0.0683	3.99E-4	0.0677	0.0151, 0.1672	31316.1141	28739.2
**k**	**8.5291**	**0.0331**	**1.5752**	**5.8431, 11.6174**	**3.9756E5**	**2263.8**
**xo**	**0.7846**	**1.05E-3**	**0.0556**	**0.6942, 0.9074**	**3.2109E5**	**2803**
α	1.2539	8.53E-3	0.6178	0.2431, 2.4652	1.7171E5	5241.3

β	27.2065	0.2485	15.6739	1.0471, 56.301	2.2615E5	3979.6

Supplemental Table S3. Raw data obtained from Tracer (Rambaut *et al*., 2014) for ferns' assimilation rate (A). Four species were input in the model for estimating the overall effect of light, vapor pressure deficit (VPD) and circadian regulation (time), with independent intercepts (μ) and random error (σ) for each species. The hyper-priors for the random errors were α and β. Time is described by *k* and *xo* that are the parameters of a logistic function. The model had 1.000M iterations, but saving 1 each 100, and a burn-in of 10M.

**Variable**	**Mean**	**SE**	**SD**	**95% HPD int**	**ACT**	**ESS**
Posterior	3789.9495	0.058	2.8157	3784.20, 3794.54	3.8145E5	2359.4
Likelihood	3800.8664	0.049	2.3908	3796.05, 3805,21	3.7783E5	2382
Prior	-10.917	0.0323	1.6378	-14.193, -8.7671	3.5091E5	2564.8
μ1	0.5127	0.0000575	0.00274	0.5077, 0.5184	3.9602E5	2272.6
μ2	0.6512	0.0000862	0.00416	0.6427, 0.6592	3.8691E5	2326.1
μ3	0.5453	0.0000706	0.00338	0.5385, 0.5578	3.935E5	2287.2
μ4	0.5419	0.0000658	0.00325	0.5356, 0.5482	3.9312E5	2289.4
σ1	0.0195	0.0000194	0.000937	0.0182, 0.0214	3.8749E5	2322.6
σ2	0.0608	0.0000432	0.000211	0.0575, 0.0657	3.7741E5	2384.7
σ3	0.0368	0.0000245	0.001197	0.0343, 0.039	3.7813E5	2380.2
σ4	0.0334	0.0000216	0.00105	0.0312, 0.0353	3.7874E5	2376.3
**Light**	**0.3289**	**0.000239**	**0.0114**	**0.3074, 0.3517**	**3.924E5**	**2293.5**
**VPD**	***0.00346***	***0.000102***	***0.00487***	***-0.0055, 0.0128***	***3.9649E5***	***2269.9***
**time**	**0.1952**	**0.000122**	**0.0058**	**0.1829, 0.2045**	**3.9745E5**	**2264.5**
σ-A	0.3737	0.004189	0.2272	0.1277, 0.8634	3.0587E5	2942.4
k	***-0.0702***	***0.000971***	***0.0482***	***-0.1664, 0.015***	***3.6454E5***	***2468.8***
**xo**	**0.747**	**0.003548**	**0.1686**	**0.4027, 0.9896**	**3.9858E5**	**2258**
α	1.6486	0.0169	0.8158	0.3821, 3.358	3.84E5	2343.7

β	31.1016	0.3546	17.1725	3.3886, 61.7826	3.837E5	2345.6

Supplemental Table S4. Raw data obtained from Tracer (Rambaut *et al*., 2014) for angiosperms' assimilation rate (A). Four species were input in the model for estimating the overall effect of light, vapor pressure deficit (VPD) and circadian regulation (time), with independent intercepts (μ) and random error (σ) for each species. The hyper-priors for the random errors were α and β. Time is described by *k* and *xo* that are the parameters of a logistic function. The model had 1.000M iterations, but saving 1 each 100, and a burn-in of 10M.

**Variable**	**Mean**	**SE**	**SD**	**95% HPD int**	**ACT**	**ESS**
Posterior	2557.8101	0.0572	2.8677	2551.74, 2562.29	3.5867E5	2509.3
Likelihood	2571.4631	0.0468	2.3741	2566.74, 2575.31	3.493E5	2576.6
Prior	-13.653	0.0283	1.5661	-16.83, -11.529	2.935E5	3066.5
µ1	0.5224	0.00035	0.0168	0.4903, 0.5518	3.9645E5	2270.1
µ2	0.5001	0.00034	0.0164	0.473, 0.5335	3.9772E5	2262.9
µ3	0.5235	0.00034	0.0164	0.4935, 0.5524	3.9858E5	2258
µ4	0.4706	0.00035	0.0168	0.4413, 0.5014	3.9626E5	2271.3
σ1	0.0696	0.00005	0.0026	0.0646, 0.0745	3.3203E5	2710.6
σ2	0.0641	0.00005	0.0023	0.0593, 0.0685	3.5773E5	2515.9
σ3	0.0309	0.00003	0.0012	0.0287, 0.0333	3.5304E5	2549.3
σ4	0.0857	0.00005	0.0025	0.0808, 0.0902	3.3536E5	2683.6
**Light**	**0.4516**	**0.000415**	**0.0201**	**0.415, 0.4964**	**3.8287E5**	**2350.7**
**VPD**	**-0.1764**	**0.00018**	**0.0088**	**-0.192, -0.1613**	**3.9403E5**	**2284.1**
**time**	**0.3863**	**0.00076**	**0.0362**	**0.3226, 0.4565**	**3.9893E5**	**2256**
σ-A	0.5533	0.00512	0.3238	0.1681, 1.1992	2.256E5	3989.3
k	**-0.1874**	**0.00116**	**0.0594**	**-0.2998, -0.0735**	**3.4494E5**	**2609.2**
**xo**	**0.4611**	**0.0064**	**0.3041**	**0.00739, 0.9484**	**3.9892E5**	**2256.1**
α	2.1478	0.0216	1.0723	0.364, 4.1387	3.6653E5	2455.4

β	27.5171	0.3044	15.0842	3.3284, 58.0822	3.6652E5	2455.5

30

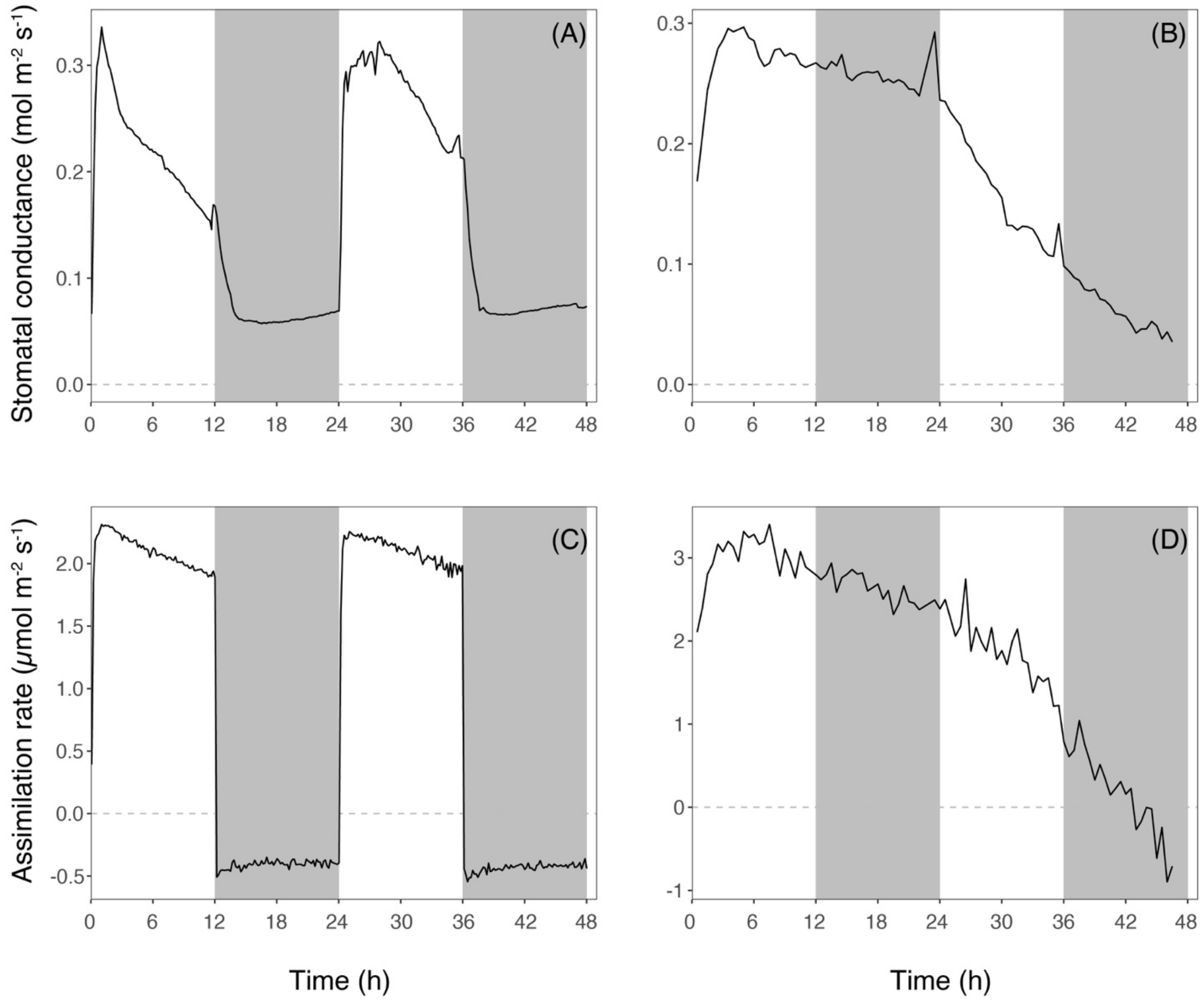

Supplemental Figure S1. Gas exchange measurements for a Flowering fern (*Anemia phyllitidis)* under diel light/dark cycles versus constant light. (A-B) Stomatal conductance and (C-D) photosynthesis over 48 h. Plants were acclimated to 12 h dark/light cycle for (A&C) dial measurement with true days and nights, and (B&D) measurements under constant light for 48 h. The gray areas indicate (subjective-) nights and the white areas indicate (subjective-) days.

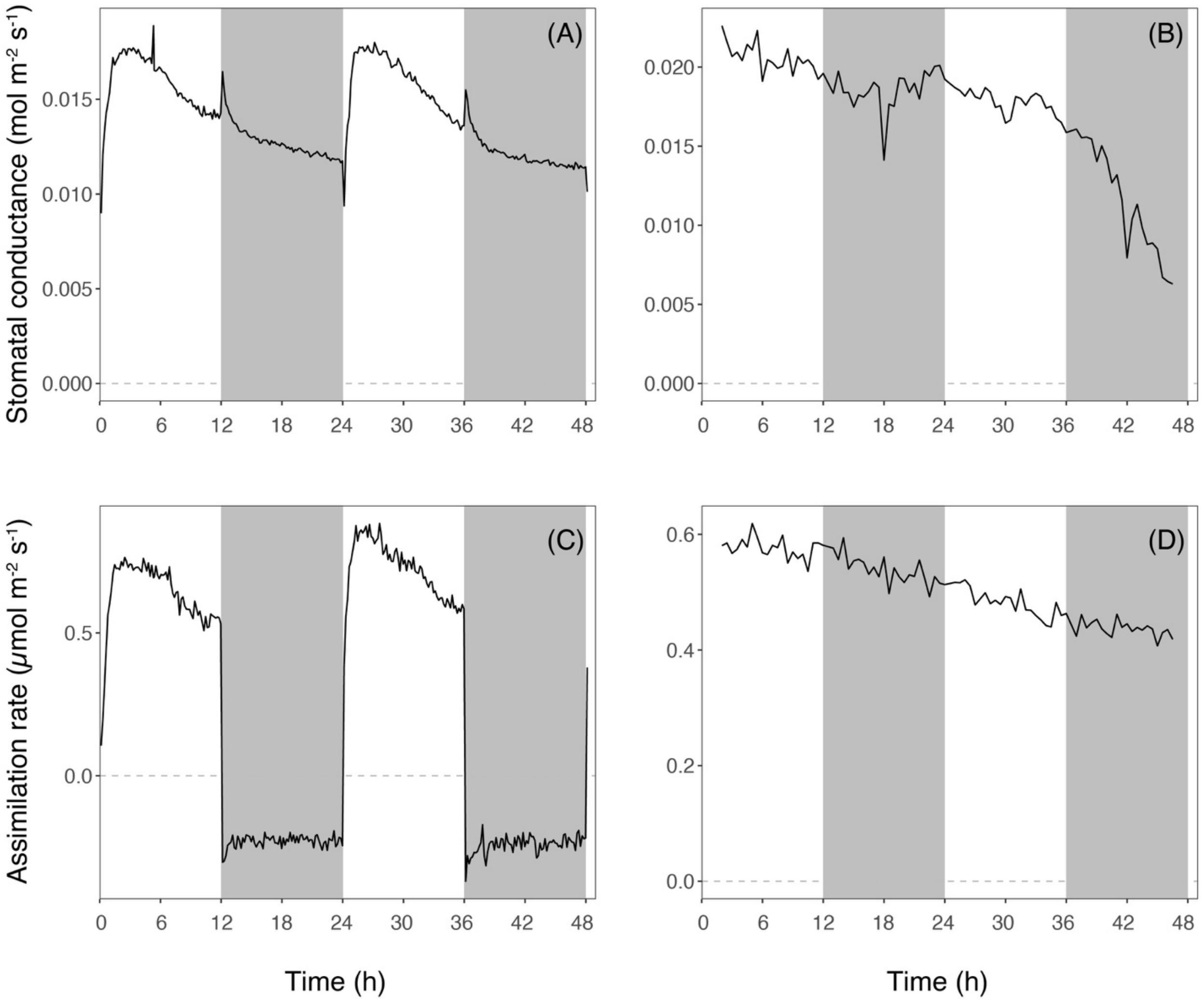

Supplemental Figure S2. Gas exchange measurements for a Spleenwort fern (*Asplenium inaequilaterale)* under diel light/dark cycles versus constant light. (A-B) Stomatal conductance and (C-D) photosynthesis over 48 h. Plants were acclimated to 12 h dark/light cycle for (A&C) dial measurement with true days and nights, and (B&D) measurements under constant light for 48 h. The gray areas indicate (subjective-) nights and the white areas indicate (subjective-) days.

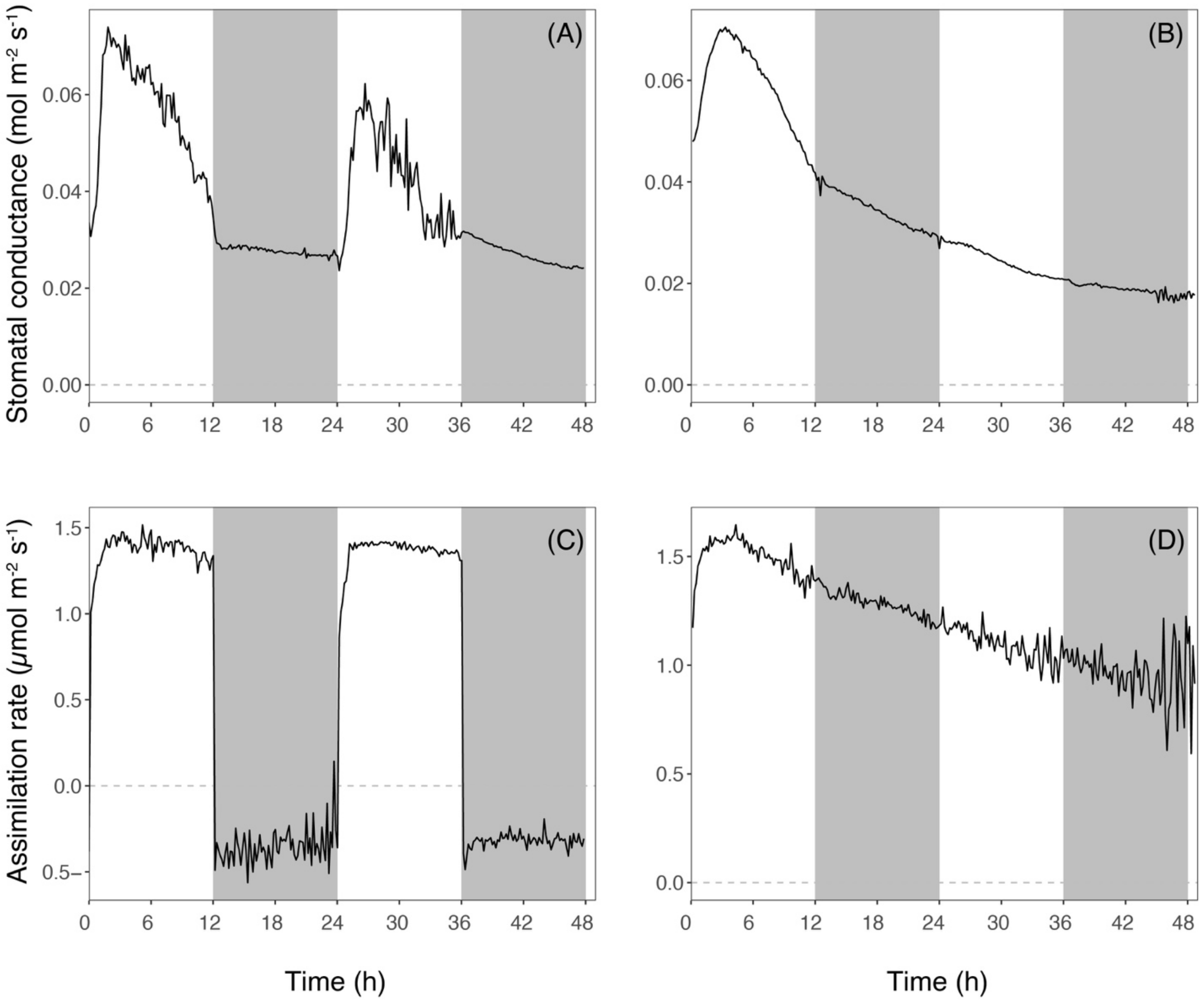

Supplemental Figure S3. Gas exchange measurements for a Water sprite (*Ceratopteris thalictroides)* under diel light/dark cycles versus constant light. (A-B) Stomatal conductance and (C-D) photosynthesis over 48 h. Plants were acclimated to 12 h dark/light cycle for (A&C) dial measurement with true days and nights, and (B&D) measurements under constant light for 48 h. The gray areas indicate (subjective-) nights and the white areas indicate (subjective-) days.

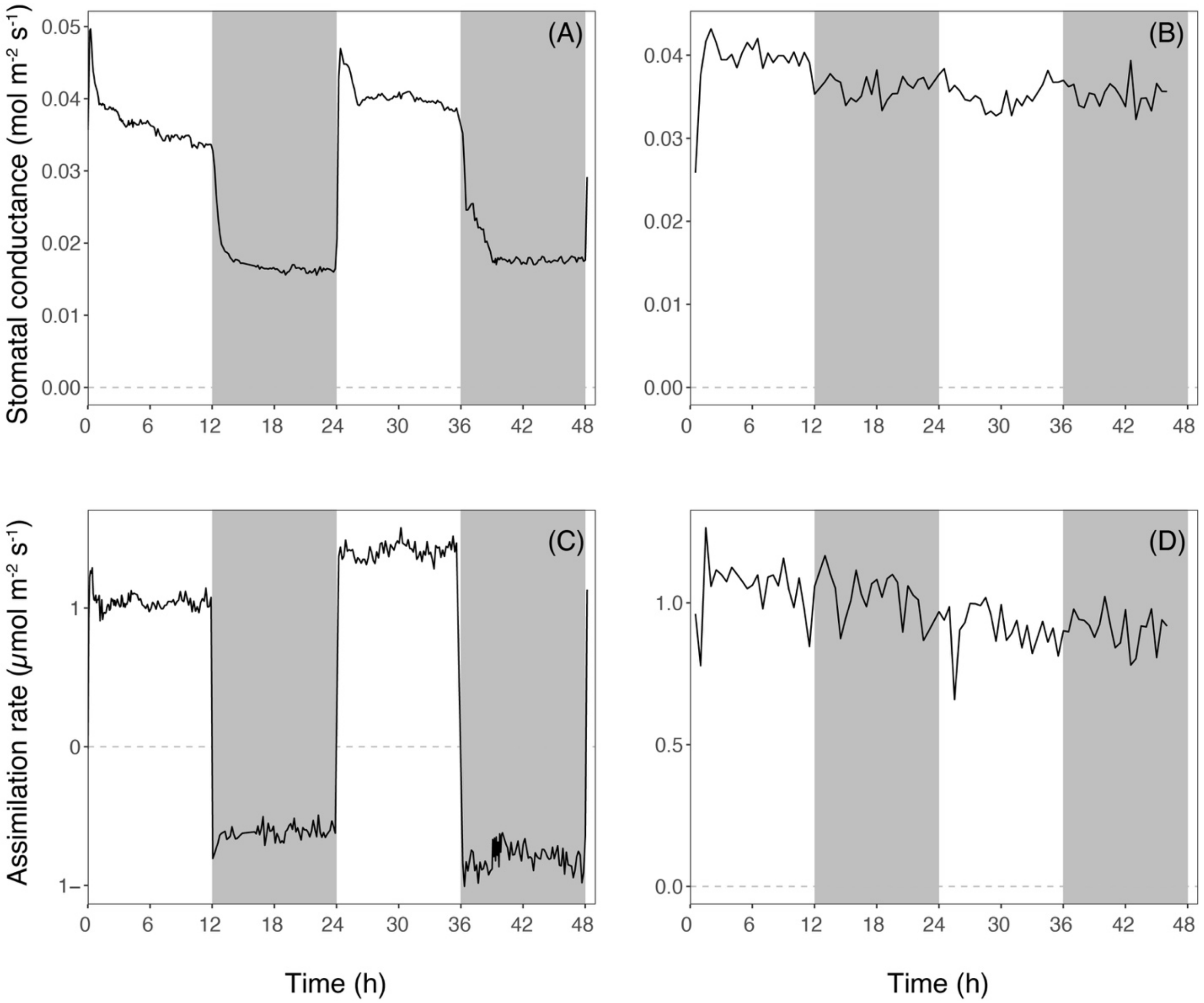

Supplemental Figure S4. Gas exchange measurements for an Adders fern (*Polypodium vulgare* under diel light/dark cycles versus constant light. (A-B) Stomatal conductance and (C-D) photosynthesis over 48 h. Plants were acclimated to 12 h dark/light cycle for (A&C) dial measurement with true days and nights, and (B&D) measurements under constant light for 48 h. The gray areas indicate (subjective-) nights and the white areas indicate (subjective-) days.

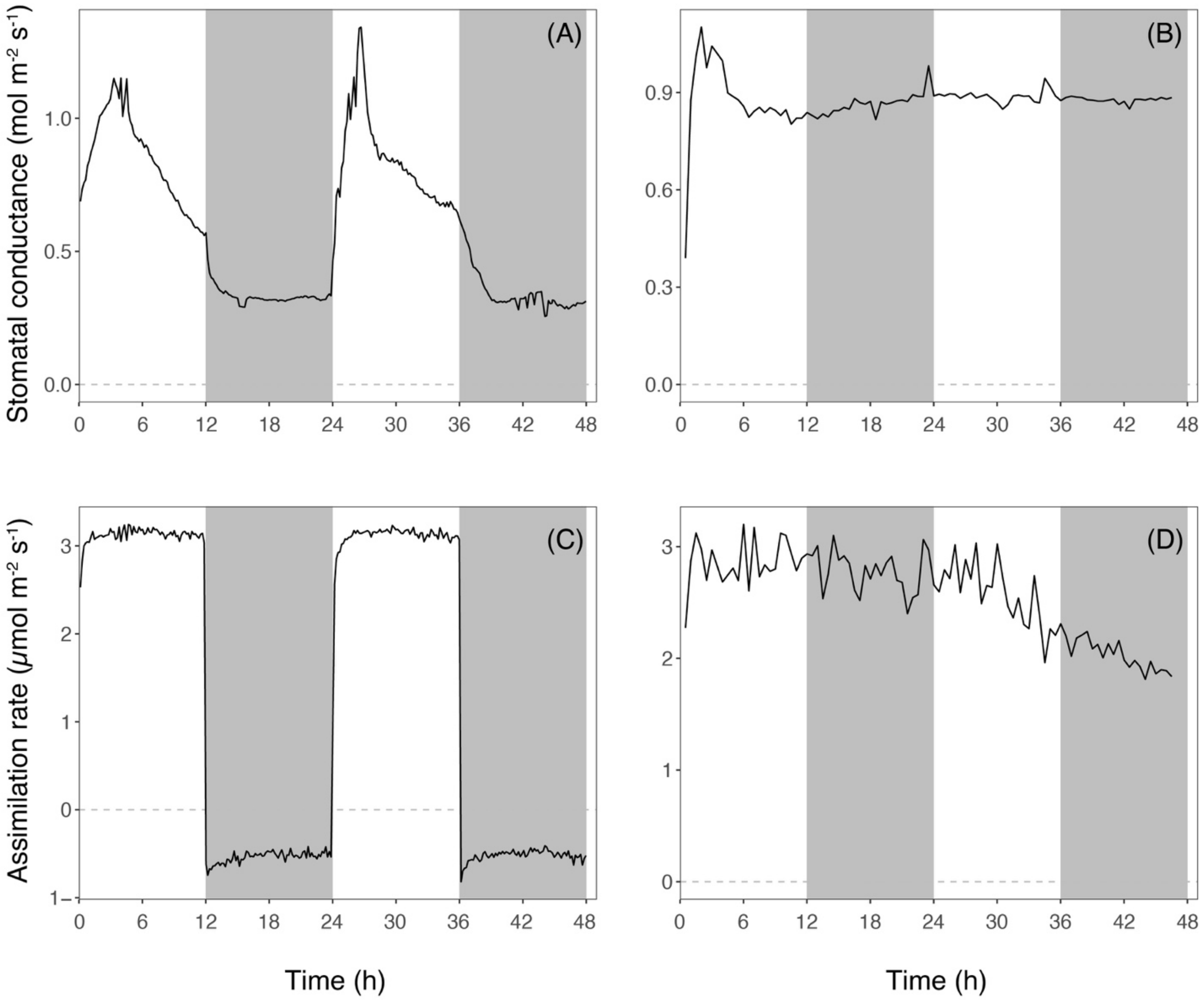

Supplemental Figure S5. Gas exchange measurements for a Spike-moss (*Selaginella tamariscina)* under diel light/dark cycles versus constant light. (A-B) Stomatal conductance and (C-D) photosynthesis over 48 h. Plants were acclimated to 12 h dark/light cycle for (A&C) dial measurement with true days and nights, and (B&D) measurements under constant light for 48 h. The gray areas indicate (subjective-) nights and the white areas indicate (subjective-) days.

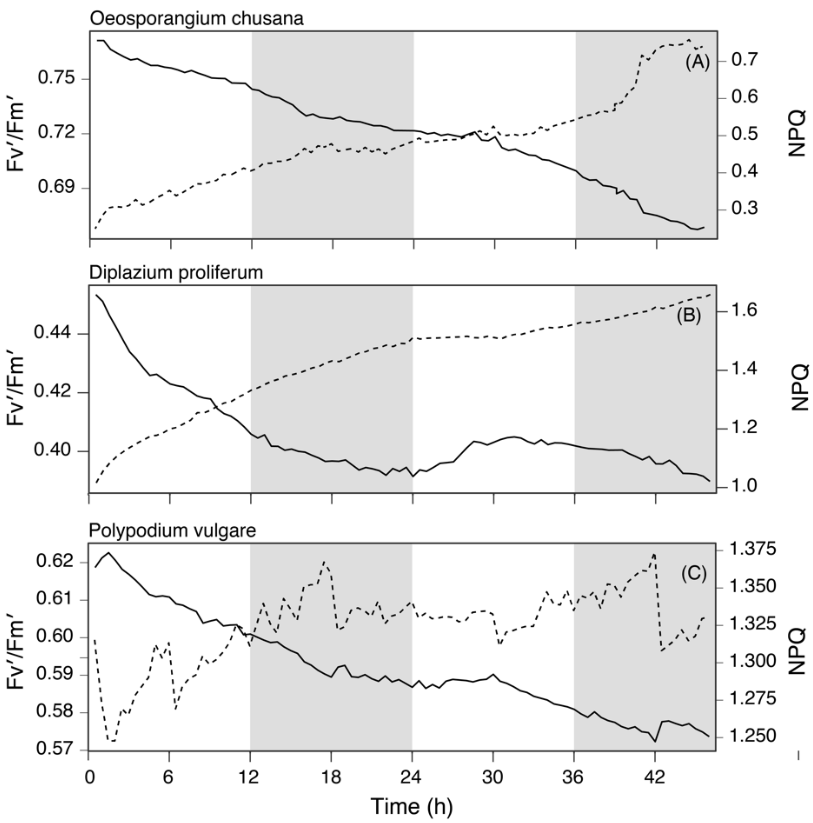

Supplemental Figure S6. Photosynthetic reaction centre rhythm of three fern species over 46 hours of constant light. Photosystem II operating efficiency (*Fv'/Fm'*) indicated with a solid line, and non-photochemical quenching (*NPQ*) with a dashed line of (A) *Oeosporangium chusana*, (B) *Diplazium proliferum*, and (C) *Polypodium vulgare*. The gray areas indicate (subjective-) nights and the white areas indicate (subjective-) days.

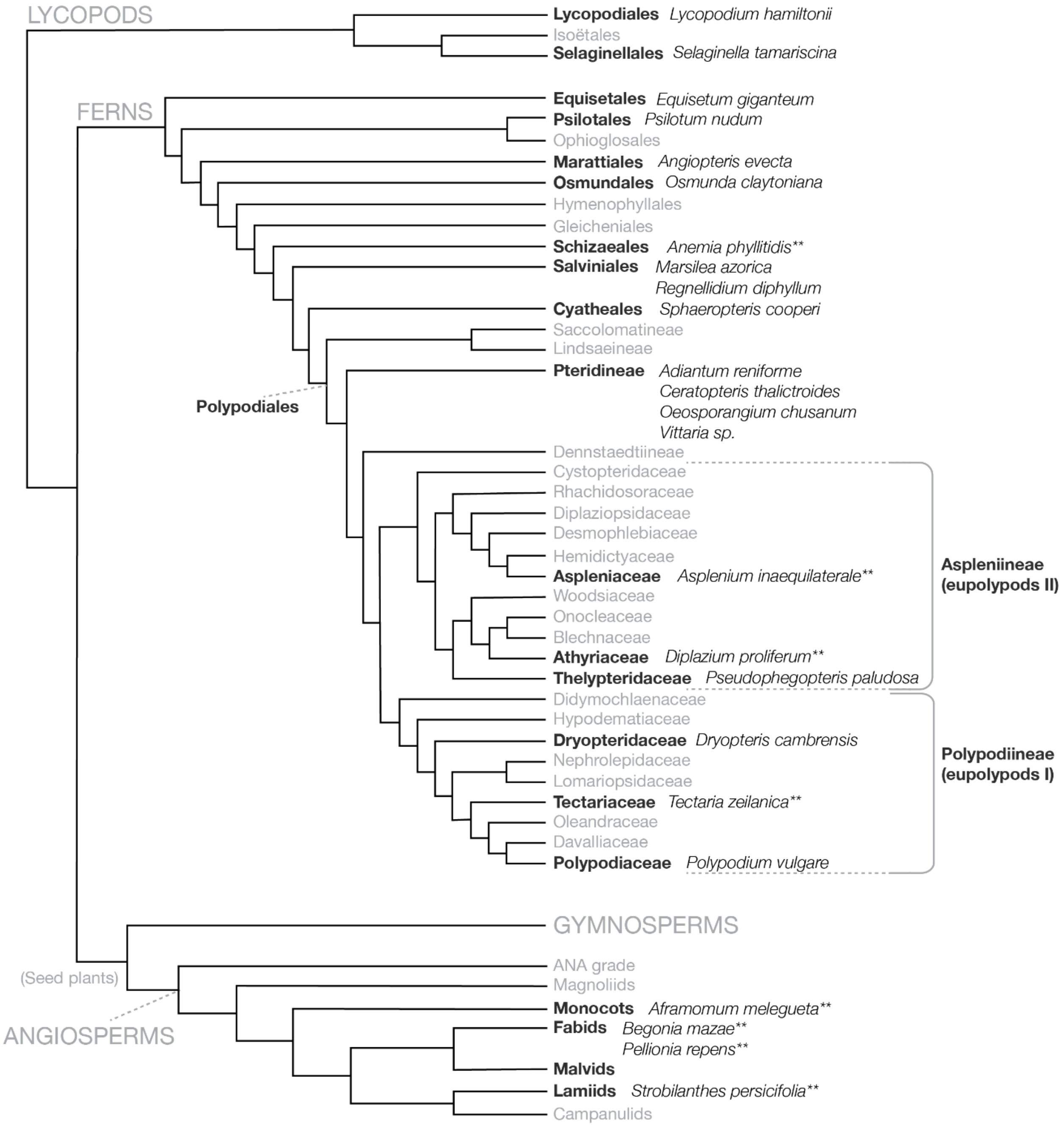

Supplemental Figure S7. Phylogeny of vascular plants used in this study. In bold and black are highlighted all the groups included in the study of circadian rhythms, while in gray the groups not included. All named ferns species were used in the growing chamber experiment. ** are indicating the species used in the greenhouse experiment. Adapted from PGG I (2016) fern phylogeny, and Cole, Hilger and Stevens (2019) angiosperm phylogeny poster.

References

Cole, T.C.H., Hilger, H.H., Stevens, P., 2019. Angiosperm phylogeny poster (APP) – Flowering plant systematics, 2019 (No. e2320v6). PeerJ Inc. https://doi.org/10.7287/peerj.preprints.2320v6

PPG I, 2016. A community-derived classification for extant lycophytes and ferns. Journal of Systematics and Evolution 54, 563–603. https://doi.org/10.1111/jse.12229

Rambaut, A., Drummond, A., Xie, D., Suchard, M., 2014. Tracer v1.6.
